# Massive Localized Abdominal Lymphedema: A Case Report with Literature Review

**DOI:** 10.1055/a-2140-8589

**Published:** 2023-11-30

**Authors:** Badri Gogia, Irina Chekmareva, Anastasiia Leonova, Rifat Alyautdinov, Grigory Karmazanovsky, Andrey Glotov, Dmitry Kalinin

**Affiliations:** 1 Department of Herniology and Plastic Surgery, A.V. Vishnevsky National Medical Research Center of Surgery, Moscow, Russia; 2 Department of Morbid Anatomy, A.V. Vishnevsky National Medical Research Center of Surgery, Moscow, Russia; 3 Department of the Interventional Endoscopy, A.V. Vishnevsky National Medical Research Center of Surgery, Moscow, Russia; 4 Department of Radiology and Magnetic Resonance Imaging, A.V. Vishnevsky National Medical Research Center of Surgery, Moscow, Russia; 5 Department of Morbid Anatomy, A.V. Vishnevsky National Medical Research Center of Surgery, Moscow, Russia

**Keywords:** massive localized lymphedema, pseudosarcoma, soft tissue tumor, obesity, morphological study, Introduction

## Abstract

Massive localized lymphedema (MLL) is a rare disease caused by the obstruction of lymphatic vessels with specific clinical morphological and radiological characteristics. People with morbid obesity are mainly affected by MLL. Lymphedema is easily confused with soft tissue sarcoma and requires differential diagnosis, both the possibility of an MLL and also carcinoma manifestations in the soft tissues. The possible causes of massive lymphedema include trauma, surgery, and hypothyroidism. This report is the first case of MLL treated surgically in the Russian Federation. Detailed computed tomography (CT) characteristics and an electron microscope picture of MLL are discussed. A 50-year-old woman (body mass index of 43 kg/m ^2^ ) with MLL arising from the anterior abdominal wall was admitted to the hospital for surgical treatment. Its mass was 22.16 kg. A morphological study of the resected mass confirmed the diagnosis of MLL. We review etiology, clinical presentation, diagnosis, and treatment of MLL. We also performed an electron-microscopic study that revealed interstitial Cajal-like cells telocytes not previously described in MLL cases. We did not find similar findings in the literature. It is possible that the conduction of an ultrastructural examination of MLL tissue samples will further contribute to the understanding of MLL pathogenesis.

## Introduction

Massive localized lymphedema (MLL) is a rare benign soft tissue lesion that develops in morbidly obese patients. This pseudotumor lesion is caused by obstruction of lymphatic channels. MLL is sometimes called pseudosarcoma due to some macroscopic resemblance to soft tissue sarcomas. 1 2

MLL is characterized by slow growth, and more often affects females. Patients often seek medical assistance during the late stages of the disease, when daily activity is compromised. According to the literature data, the size of MLL may reach 62 cm at the time of patient admission. 3

Farshid and Weiss first described MLL as an enlarging lesion due to chronic lymph obstruction; 4 hence, other terms were also proposed. 5 MLL is commonly localized on the inner surface of the thigh, followed by the anterior abdominal wall. 6 7 8

Morbid obesity and metabolic syndrome result in the obstruction of the lymphatic vessels, ischemia, and proliferation of connective tissue. Posttraumatic damage to the lymphatic vessels can also contribute to the development of lymphedema. 9

MLL has its own clinical, radiological, and histopathological characteristics ensuring the diagnosis of the obstruction of lymphatic vessels. The aim of our report is to present the case of anterior abdominal wall MLL and discuss its clinical signs and potential diagnostic methods, including morphological ones that can be considered while treating this group of patients. It is the first time when electron-microscopic (EM) study of biopsy specimens revealed the presence of interstitial Cajal-like cells (ICLC) in MLL.

## Case

**Fig. 1:**
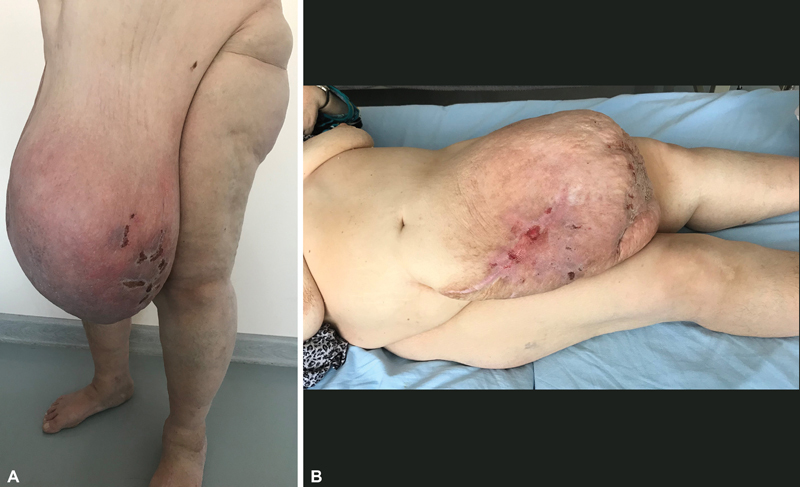
Preoperative view of the patient with a massive local lymphedema of the anterior abdominal wall. ( **A** ) Semilateral view. A patient is standing. ( **B** ) Lateral view. A patient is supine.

**Fig. 2:**
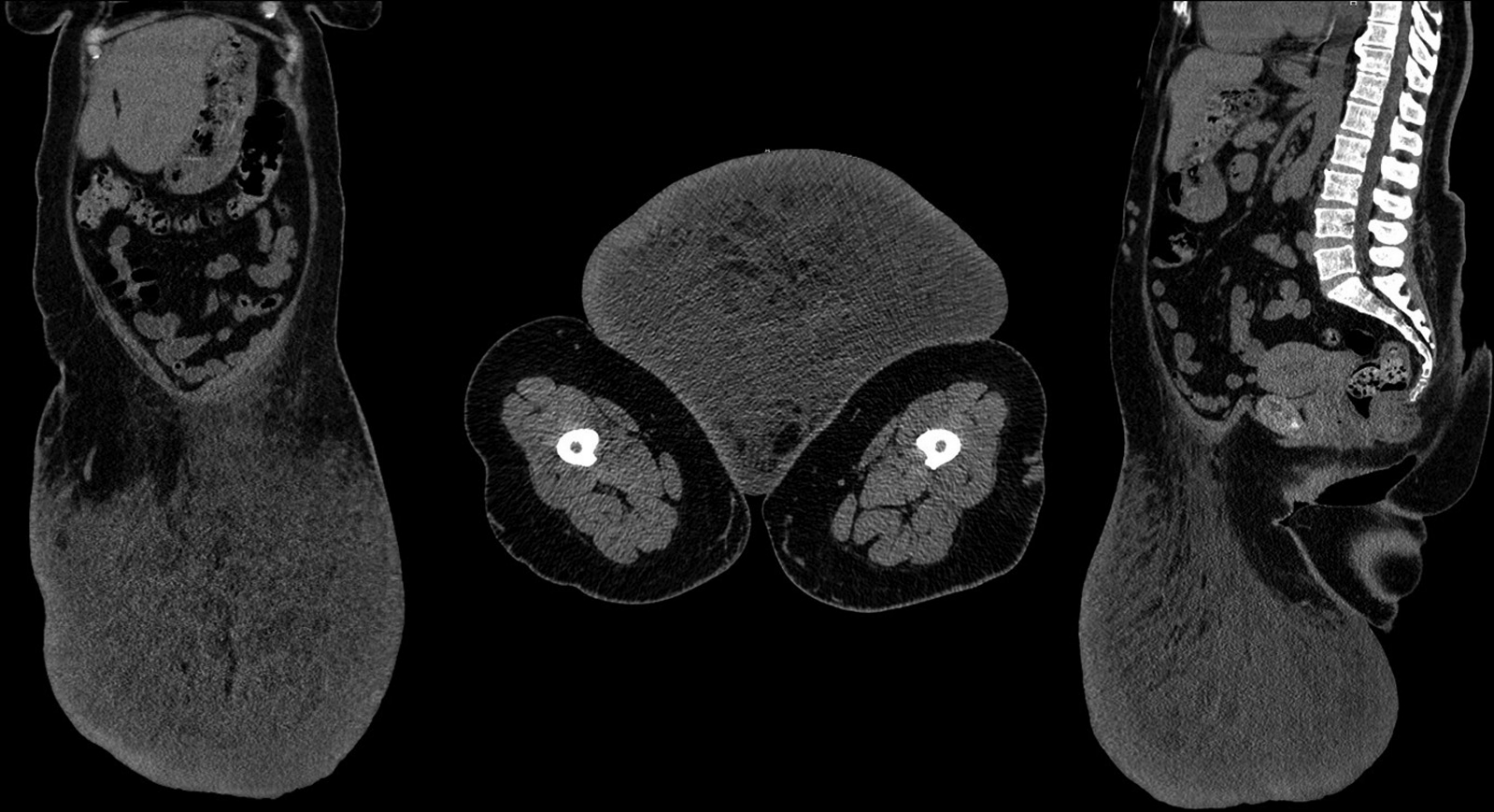
CT-scan of the anterior abdominal wall MLL.

A 50-year-old female was admitted to the Department of Plastic Surgery and Herniology of our institution in February 2019 with a mass on the anterior abdominal wall extending to the knees. The patient was morbidly obese with body mass index (BMI) of 62.5 kg/m ^2^ at the age of 47 years. In 2016 after a stroke, she lost 44 kg of body mass. She started to notice a mass in her hypogastric area in the form of an “apron” that had flesh color and moderate density. This mass gradually started to grow, the density became more pronounced, the skin surface was granular, the color became pink, and then cyanotic. Skin started to itch and trophic changes with ulcerations and small hemorrhages appeared ( Fig. 1 ). The patient had difficulty walking and problems with sleep. Computed tomography (CT) revealed a mass of the anterior abdominal wall in the hypogastric area, 27.5 cm × 31.4 cm × 33.1 cm in size with well-defined borders ( Fig. 2 ). Dilated, tortuous venous collaterals were visualized in the mass (–9.2 HU). The skin was of uniform density and was thickened evenly. The subcutaneous adipose tissue had a typical cellular structure due to longitudinal and radial fibrous cords. The density of adipose tissue in the cells increased toward the skin (11 HU). There were no other changes in the skin and subcutaneous tissue. All these features were suggestive for benign changes, the absence of sarcoma so recommended surgical intervention followed by complex pathological examination.

**Fig. 3:**
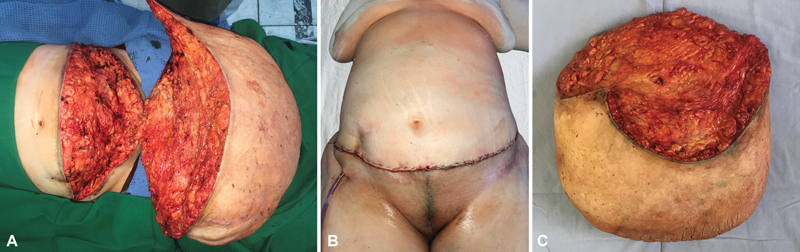
Intraoperative photos. ( **A** ) Surgical access to the excised MLL. ( **B** ) View of the sutured abdominal wall after the excision of MLL. ( **C** ) Excised specimen.

The patient underwent the anterior abdominal wall MLL resection ( Fig. 3 ). The abnormal tissue was separated from aponeurosis and was removed. There was bleeding from vessels, which were later ligated. The removed mass was 50 cm × 60 cm in the size. The weight of the mass was 22.16 kg.

**Fig. 4:**
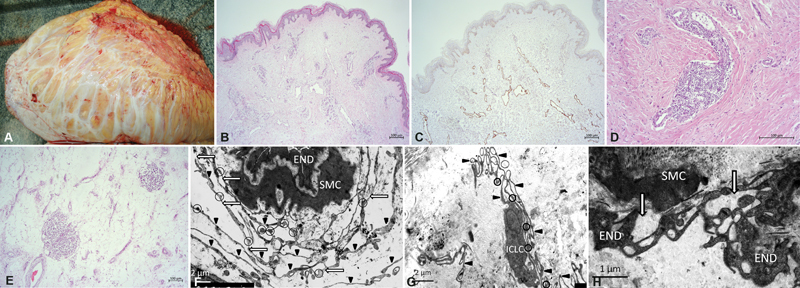
Morphological view of the MLL. ( **A** ) Macropreparation (50 × 60 × 20 cm) **—** view of the cut section: the dermis is sharply thickened, there are marked fibrous septa in the subcutaneous tissue. ( **B** ) Microscopical study (hematoxylin and eosin, ×50 magnification). Epidermis with hyperkeratosis. Dermal fibrosis and hyalinosis, multiple, slightly enlarged lymphatic vessels. ( **C** ) Immunohistochemical (IHC) staining with Podoplanin (D2-40, ×50 magnification). Positive reaction in lymphatic vessels. ( **D** ) Microscopical study (hematoxylin & eosin, ×100 magnification). Dilated lymphatic vessels with perivascular lymphoplasmacytic infiltration in the fibrous thickened dermis. ( **E** ) Microscopical study (hematoxylin & eosin, ×50 magnification).Subcutaneous tissue consists of mature adipocytes with prominent edema, presence of fibrous septa, lymphoplasmacytic infiltrate, reactive proliferation of capillaries. ( **F** ) Transmission electron microscope picture (scale bar: 2 μm) of the dermal blood vessel segment surrounded by interstitial Cajal-like cells (ICLCs) projections. A telepode—a triangle, a podomere—an arrow, a podom—a circle, endothelial cell—END, smooth muscle cell—SMC. ( **G** ) Transmission electron microscopy (scale bar: 2 μm) of the ICLC in the dermal segment. Long thin telepodes—a triangle; a podom—a circle. ( **H** ) Transmission electron microscope picture (scale bar: 1 μm) of the dermal lymphatic vessel segment with detachment of vacuolated endothelial cells—(END) and increased subendothelial space (the white arrow); smooth muscle cell—SMC.

On the cut sections, the dermis was 5 cm in depth with a whitish interlayer. Subcutaneous tissue was edematous with few scattered blood vessels and fibrous whitish interlayer. The transparent fluid was visible on the edges of the cut section ( Fig. 4A ). Histological examination revealed a morphological picture of MLL ( Fig. 4B–E ). The epidermis was with hyperkeratosis, dermis was fibrosis, dilated lymphatic vessels, and infiltrated with reactive proliferative fibroblasts in the ulceration zone. The subcutaneous tissue was composed of mature adipose tissue with marked diffuse swelling and the presence of wide septa and multiple capillaries with perivascular lymphoplasmacytic infiltration. There were no atypical adipocytes.

An EM examination of the obtained tissue sample was performed ( Fig. 4F–H ). A specimen of 1 mm ^3^ in size was cut, fixed in 2.5% glutaraldehyde and 1% osmium oxide (VIII). Then, the specimen was dehydrated using increasing concentrations of alcohol (50, 70, 96, and 100%) and impregnated with a mixture of propylene oxide and araldite resin. After impregnation, the specimen was transferred to capsules and filled with araldite resin, and then placed in a thermostat at a temperature of 60°C for 2 days. The images of light-optical examination were analyzed and recorded (section thickness of 1.0–1.5 µm, stained with toluidine blue), including the area targeting for ultratomy. The ultrathin sections of 100 to 120 nm in thickness were cut using an LKB ultramicrotome (Sweden). Sections were stained with uranyl acetate and lead citrate and viewed under a JEM 100-CX electron microscope (JEOL, Japan) in the transmission mode at an accelerating voltage of 80 kV.

ICLCs—telocytes were found in the dermis and subcutaneous tissue. These cells have not been previously described in MLL. Multiple projections of ICLCs interacted with smooth muscle cells (SMCs) of blood vessels ( Fig. 4F ). ICLCs had characteristic ultrastructural features: several prolongations—telopodes, thin segments—podomers, and dilated segments—podoms ( Fig. 4G ). There were single ICLCs around lymphatic vessels with pronounced destructive changes, but more often ICLCs were completely absent. Ultrastructural changes in ICLCs are associated with the dysfunction of these cells. The ultrastructural analysis of the walls of the lymphatic vessels showed the impairment of myendothelial and myo-myocytic contacts, as well as collagenization of the vascular walls, detachment of endothelium with fragmentation, enlargement of the subendothelial space, destruction of endothelial, and SMC organelles ( Fig. 4H ).

**Fig. 5:**
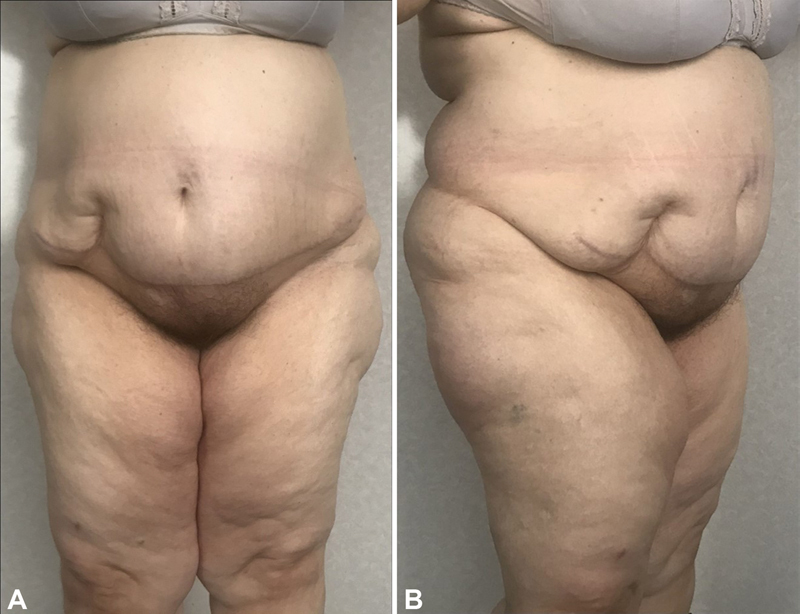
The view of the patient 1 year after the excision of MLL. ( **A** ) Anterior view. ( **B** ) Lateral view.

There were no complications after surgery. The in-hospital length of stay was 10 days. Upon follow-up in the ^1st^ year, the patient did not have any complaints. No relapses were observed ( Fig. 5 ). The quality of life significantly improved. BMI was 36.7 kg/m ^2^ .

## Discussion

The first cases of MLL as a distinct disease were described by Farshid and Weiss in 1998 after the investigation of tissue samples from 14 patients with morbid obesity. Based on clinico-morphological similarities with diffuse lymphedema, the authors called this process a MLL. 4

Kohli et al described three similar clinical cases in 2013 and gave them a new name - «Obesity-Associated Abdominal Elephantiasis» 5 .

There are no statistically proven data about the prevalence of this pathology. The main information is presented in the form of clinical cases. 6 7 Kurt et al reported a case series of MLL in 2016. In total, 54 cases of MLL were found in 46 patients undergoing treatment in the years 2002 to 2015. 8

The thigh is the most common location. Other sites were also described, including the abdominal wall, pelvic area, vulva, penis, popliteal fossa, and an upper extremity. 6 7 8

The main risk factors are morbid obesity and metabolic syndrome. 8 An increased amount of adipose tissue leads to an obstruction of lymphatic vessels of the skin and subcutaneous tissue with the development of lymphedema, ischemia, and increased deposition of connective tissue. Less common causes are trauma and surgical intervention with damage to lymphatic vessels. Hypothyroidism also was reported as a risk factor. 9

The life quality of patients with MLL is significantly decreased due to immobility, social isolation, difficulties with finding the appropriate size of clothes, and personal hygiene. Successful rehabilitation may be performed after the surgical treatment. However, the surgical method of the treatment can be complicated by difficulties in identifying the margins of the pathological tissue, increased bleeding, and lymphorrhea. 10

It is important to note that MLL is a clinical diagnosis. A preoperative biopsy is not required and is rarely informative. 11 It requires multislice spiral computed tomography or magnetic resonance imaging to exclude soft tissue sarcoma. 12 13 In the reported case, a CT study was also done to exclude the diagnosis of the anterior abdominal wall hernia and malignant mass.

Shon et al consider lymphedema as a significant risk factor for the development of angiosarcoma. 14 In total, 65 cases of MLL were described by 2015, and 9 (10.3%) of them progressed to angiosarcoma. 15 According to Best and coauthors, MLL is a benign tumor, the excision of which is desirable because it can progress to angiosarcoma in 13%. 16 Other authors also noted the malignization of MLL. 11 14 The mortality rate from MLL is 9% that justifies oncological alertness while diagnosing MLL. 13 14 17

Early diagnosis of MLL in obese patients remains challenging. In our case, the lymphedema was only visualized after the decreased subcutaneous adipose tissue despite lymphatic obstruction began to progress earlier. The lack of data for previous trauma, surgery, and the presence of hypothyroidism confirm morbid obesity as the leading cause for developing MLL in our patient. We excluded the likelihood of a malign process based on the specific changes in the skin and subcutaneous adipose tissue according to CT scans. The histological report has a significant role in the diagnosis of MLL. The determining point is a histological confirmation which together with above-mentioned findings gives the final diagnosis of MLL.

The etiology and pathogenesis of MLL is unclear but might be multifactorial. It might be assumed that the destruction of ICLCs, first identified by electron microscopy, decrease in their number or complete absence, and dysfunction of intercellular contacts are factors that can influence the initiation and the development of MLL and promote lymphatic stasis via decreasing the tone of lymphatic vessels. ICLCs are involved in cell-to-cell communication. They form a complex three-dimensional extracellular network via homocellular and/or heterocellular contacts with endothelial cells, SMCs, and activated immunocytes. ICLCs coordinate long-distance intercellular connectivity. 18 19 Some authors consider ICLCs and SMCs interactions as one of the mechanisms that explain the peristaltic contraction of lymphatic vessels. 20 Most likely, it is a complex signaling pathway that explains the role of the three-dimensional extracellular network in the regulation of contractile activity of lymphatic vessels. Decreased telocyte numbers might be an important etiopathogenic factor in the development of MLL.

Thus, we may conclude that MLL is a complication of obesity that significantly reduces the quality of life and requires surgical treatment according to the literature analysis and our experience. It has its own clinical and instrumental features that allow diagnosing this rare disease.
